# Physical activity measurement in older adults: Wearables versus self-report

**DOI:** 10.3389/fdgth.2022.869790

**Published:** 2022-08-31

**Authors:** Anna VandeBunte, Eva Gontrum, Lauren Goldberger, Corrina Fonseca, Nina Djukic, Michelle You, Joel H. Kramer, Kaitlin B. Casaletto

**Affiliations:** ^1^ Department of Neurology, Memory and Aging Center, University of California, San Francisco, San Francisco, CA, United States; ^2^ Department of Psychology, Palo Alto University, Palo Alto, CA, United States

**Keywords:** healthy aging, fitbit, actigraphy, CHAMPS, PASE

## Abstract

Physical activity (PA) is associated with preserved age-related body and brain health. However, PA quantification can vary. Commercial-grade wearable monitors are objective, low burden tools to capture PA but are less well validated in older adults. Self-report PA questionnaires are widely accepted and more frequently used but carry inherent limitations. We aimed to compare these commonly used PA measures against one another and examine their convergent validity with a host of relevant outcomes. We also examined the factors that drive differences in PA self-reporting styles in older adults. 179 older adults completed 30-day Fitbit Flex2™ monitoring and reported PA levels *via* two widely used PA questionnaires: PASE and CHAMPS-METs (metabolic expenditure calories burned). Participants also completed measures of cardiometabolic (hypertension diagnosis, resting heart rate, A1C levels), cognitive (memory, processing speed, executive functioning), and brain MRI (medial temporal lobe volume) outcomes. The discrepancy between objective Fitbit monitoring and self-reported PA was evaluated using a sample-based z difference score. There were only modest relationships across all PA metrics. Fitbit step count demonstrated a stronger association with the PASE, whereas Fitbit calories burned was more strongly associated with CHAMPS-MET. Fitbit outcomes had more consistent convergence with relevant outcomes of interest (e.g., cardiometabolic and brain health indices) when compared to subjective measures; however, considerable heterogeneity within these associations was observed. A higher degree of overreporting was associated with worse memory and executive performances, as well as hypertension diagnoses. We build on prior findings that wearable, digital health indicators of PA demonstrate greater construct validity than self-report in older adults. We further show important clinical features (e.g., poorer cognitive status) of older adults that could contribute to a higher level of overreporting on self-report measures. Characterization of what PA measures truly operationalize will help elucidate relationships between most relevant facets of PA and outcomes of interest.

## 1. Introduction

Physical activity (PA) is associated with preserved vascular health, brain structure, and cognition with age (1–3). In the context of neurodegenerative diseases, active lifestyles are also linked with less functional decline and reduced risk of dementia (2, 4, 5). However, physical activity is a broad construct, and its operationalization can vary widely depending on the measures employed, possibly leading to imprecision and/or inconsistencies in the literature linking physical activity to brain health.

The most commonly utilized measures of PA may capture several aspects of activity. Actigraphy monitors are useful for objective assessment of free-living movement levels, ranging from everyday chores to structured exercise. Given their relative low cost and user friendly interfaces, commercially available wearables (like Fitbit actigraphy monitors) have garnered increasing attention and validation of metrics for reliable measurement of PA (6, 7). For instance, Fitbit monitors have demonstrated inter-device reliability with other actigraphy monitors (i.e., Actigraph GT3X+ accelerometer) and positive correlations with observed step count and gait speed on a treadmill (8–10). Within the Fitbit suite of outcomes, average steps has demonstrated the most robust validity though there is less validity for energy expenditure as a measurement of PA (11). However, few studies have been conducted in older adults who may have varying experience using wearable devices. As digital health tools expand, understanding their utility in populations that may be most vulnerable (e.g., older adults) is needed. Moreover, considering the importance of engaging in PA for healthy aging, it important to understand the convergence across measures, how measures of PA may differ, and what are predictors of discrepancies between measures.

In the absence of actigraphy metrics, standard self-report measures of PA are used in older adults to capture more structured activities and exercise routines (e.g., duration, type of exercise). The Community Healthy Activities Model Program for Seniors (CHAMPS) and Physical Activity Scale for the Elderly (PASE) are two such widely used self-report questionnaires of PA in older adults. However, these subjective measures may carry inherent limitations and capture only segments of PA. For instance, the PASE evaluates the level of activity (e.g., frequency and duration), whereas the primary outcome for CHAMPS is metabolic expenditure. When considering utility, each is relatively quick, well-validated, and inexpensive to use. PASE has previously been associated with portable accelerometer readings, walking steps, and energy expenditure (12, 13). Higher PASE scores (indicating a greater degree of PA) have also been correlated with reduced likelihood of cardiometabolic, neurological, and psychological health conditions (14, 15). PASE has also demonstrated meaningful associations with health status and physiologic measures, such as heart rate and static balance (13, 16). Similarly, the CHAMPS has been shown to relate to a host of relevant outcomes, such as physical functioning and psychological health (17). Similar to the PASE, the CHAMPS has demonstrated positive associations with total minutes of movement measured by an accelerometer and corresponding intensity, as well as measures of fitness capacity (i.e., the 6-min walk test) and lower body physical functioning (i.e., Short Physical Performance Battery) (17–19). However, such self-report tools may be limited by scope and subjectivity. For instance, self-report measures typically underestimate sedentary time compared to real-time digital health measures, such as an accelerometer or inclinometer (20). This is particularly important when using subjective measurements to assess older adults with cognitive difficulties, as there is greater risk of recall bias (21). Furthermore, individuals who have been encouraged to engage in exercise (e.g., many older adults by their physicians) have demonstrated the tendency to engage in more overreporting, perhaps related to well-known effects of social desirability bias (22).

To date, there is a gap in the literature directly comparing and evaluating PA as assessed across multiple standardly employed measures. Studies have not pragmatically demonstrated how metrics within the Fitbit suite compare against widely used self-report measures of PA in older adults. Similarly, research has yet to compare commonly used self-report measures (i.e., CHAMPS and PASE) alongside a comprehensive panel of relevant neurologically relevant aging outcomes (e.g., cognition, MRI outcomes) to characterize their convergent validity for use in brain aging studies. Furthermore, it is uncertain whether there are discrepancies in reporting styles across self-report measures, and if particular participant characteristics systematically predict older adults who over or under report. The current study will begin to elucidate some of these relationships to contribute to our understanding PA measurement tools in older adults.

In the current study, we aimed to (1) determine the comparability across commonly used self-reported measures of physical activity (PA) and Fitbit-based actigraphy metrics, (2) examine the convergent validity of Fitbit, PASE, and CHAMPS using a comprehensive panel of demographic, cardiometabolic, cognitive, and brain structural outcomes, and (3) examine the person-specific factors that characterize overreporting on self-report measures We hypothesized that objective measures of PA *via* Fitbit would demonstrate the best construct validity and that the degree of overreporting would relate to poorer neurobehavioral status.

## 2. Materials and methods

One hundred seventy-nine older adults enrolled in the UCSF Memory and Aging Center's Longitudinal Aging Study who choose to participate in 30-day Fitbit monitoring (average daily steps and calories burned), and who completed at least one measure of self-reported physical activity levels (PASE, *n* = 105; CHAMPS-MET, *n* = 85) were included in the study (see Table 1). Participants completed comprehensive neurological and neuropsychological evaluations, as well as structural neuroimaging, cardiometabolic measures, and a study partner interview. Following evaluations, participants were reviewed at a case conference with board certified neurologists and neuropsychologists. Inclusion criteria for enrollment consisted of: (1) no current evidence of a memory or neurological condition (e.g., stroke, epilepsy), (2) no functional decline as operationalized as a Clinical Dementia Rating (CDR) scale of 0–0.5 *via* study partner interviews, (3) no history or evidence of DSM-5 major psychiatric disorders, active substance abuse, hepatitis C, blindness, deafness, HIV, and syphilis. Participants had very minimal cardiovascular medical histories (Myocardial Infarction, *n* = 4, Cerebrovascular Accident, *n* = 2, Transient Ischemic Attack, *n* = 2; note, all cardiovascular events occurred >5 years prior to study participation). The study was approved by the institutional review board of the University of California, San Francisco and is conducted in accordance with the latest Declaration of Helsinki, including written informed consent from all participants.

**Table 1:** Descriptive statistics.

	*n*	% or *M* (*SD*)
Sex, % female	105	58.66%
Race		
White	153	85.47%
Black	2	1.12%
Asian	19	10.61%
Other	5	2.79%
Age (years)	179	73.50 (8.23)
Education (years)	179	17.57 (1.85)
Fitbit steps (daily average)	179	7840.77 (3365.11)
Fitbit calories (daily average)	179	1862.27 (426.51)
PASE (possible range 0 to >500)	105	126.10 (60.66)
CHAMPS-MET (max calories burned in a week)	85	4062.76 (2275.75)
Hypertension, % yes	116	37.93%
Resting heart rate (bpm)	165	66.62 (9.51)
Hemoglobin A1C (%, normal range 4.3–5.6)	97	5.47 (0.33)
Technology Familiarity Questionnaire (Q8)^d^	128	4.50 (0.68)
Technology Familiarity Questionnaire (Q9)^e^	128	4.86 (0.41)
Memory (z-score)^a^	124	−0.07 (0.87)
Executive functioning (z-score)^c^	132	0.77 (0.58)
Processing speed (z-score)^b^	126	−2.60 (1.58)
Medial temporal lobe volume (voxels, 1 cm^3^)	72	9.80 (1.05)

Note. *N* = 175.

^a^ z-scores on these tests represent performances compared to the larger Hillblom Aging cohort of older adults.

^b^ z-score represents performance compared to young adults (20–30 years old).

^c^ z-score derived from EXAMINER normative study group (adults aged 18–80+).

^d^ Question 8: “How much difficulty do you have using computers?” (Range 1–5, 1 = extreme difficulty, 5 = no difficulty).

^e^ Question 9: “How anxious (or nervous) do you typically feel when using a computer, tablet, or smartphone?” (Range 1–5, 1 = extremely anxious, 5 = not anxious).

### 2.1. Procedure

At the in-person visit, participants neuropsychological evaluations, brain MRI, self-reported measured, and Fitbit set-up. Participants then completed 30 days of subsequent Fitbit monitoring before mailing their device back to study personnel. All data from Fitbit devices were synced to the Fitabase platform and data were downloaded for quality control, cleaning, and analysis.

### 2.2. Actigraphy monitoring

The FitBit Flex2™ (Fitbit Inc., San Francisco, CA, USA; https://www.fitbit.com) recorded average daily steps and calories burned. The FitBit Flex2™ is a thin, flexible, Bluetooth fitness tracker with no visible record of physical activity measurements, with a three-axis acceleration sensor, and with the capability to store 7 days of detailed motion data. Participants were blinded to all notifications and indication of the duration of exercise for the 30-day time period. They were instructed to wear the Fitbit during all waking hours, and to synchronize nightly with their smartphone *via* Bluetooth 4.0 before charging at night. In cases where the participant did not have a Fitbit-compatible smartphone, the Fitbit was synchronized to an iPad and aggregate daily physical activity data was collected at the completion of the 30-day period. Fitbit accounts for each participant were connected to Fitabase, a platform specifically tailored for wearable research data management.

Average daily steps and average daily calories burned were selected as the primary objective outcomes of interest. Fitbit averages were calculated by taking the average daily steps and calories burned for the first 20 days of available monitoring data. Individual days with fewer than 100 steps were removed from the analyses to control for nonadherence. Participants were only included if they had at least 14 days of available monitoring data, and the first 20 days of the 30-day monitoring data were used in the analyses.

### 2.3. Physical activity questionnaires

#### 2.3.1. Physical activity scale for the elderly

Self-reported physical activity was measured using the Physical Activity Scale for the Elderly (PASE), a widely validated measure of self-reported activity levels for older adults. Participants were asked to rate the frequency, duration, and intensity of activity in three domains (leisure, household, and work-related activity) over the past seven days with the 11-item questionnaire. Utilizing the scoring manual, activity scores were computed by multiplying activity frequencies by the task-specific weights (16). Activity scores were then summed to obtain a total score representing overall physical activity level, with higher values indicating greater activity.

#### 2.3.2. CHAMPS-MET

The Community Healthy Activities Model Program for Seniors physical activity questionnaire (CHAMPS) was administered to assess the variety of physical activities that older adult participants may engage in, from less intensive forms such as walking or stretching to more vigorous exercise routines (17). The questionnaire includes 41 items to evaluate the frequency and duration of light, moderate, and vigorous activities that were performed weekly over the last four weeks. Participants reported whether they participated in an activity during the four-week period and then selected the hours per week spent participating in the activity, rating the duration on a six-point scale from less than 1 to 9 or more hours. Each activity corresponds to a metabolic weight or MET value. Estimated caloric expenditure was calculated by multiplying the estimated duration of each activity by the corresponding MET value, in alignment with published guidelines (17).

### 2.4. Cognitive outcomes

Participants completed a neuropsychological battery assessing cognitive outcomes hypothesized to be associated with physical activity (23, 24). This brief standardized battery has been previously described and validated to be neuroanatomically sensitive to age-related neurodegeneration (25, 26).

#### 2.4.1. Episodic memory

Verbal episodic memory was measured by the California Verbal Learning Test (CVLT-II) and a modified version of the Benson Figure Memory test. The CVLT-II includes a 16-item list presented over five learning trials, followed by free and cued recall of the list after an interference trial, and then again after a 20-min delay. Following the long delay, participants were given a list of 44 words and asked to discriminate between the target word and a distractor item (recognition trial). Outcome metrics included words correctly recalled after delays and recognition discrimination performance.

To assess visual memory, participants were asked to draw the modified Benson figure from memory after a 10-min delay. Recall of the figure was scored on a 17-point scale (25).

Sample-based z-scores were created for outcomes on both measures and averaged together to create an episodic memory composite.

#### 2.4.2. Processing speed

Processing speed was assessed through five computerized tests of reaction time to different visual stimuli (dots, lines, search, shapes, abstract matching 1, abstract matching 2) (27). All tasks included a practice trial period where the participant had to perform at greater than 70% accuracy in order to continue to the test trials. Sample-based z-scores were created for each of the five tasks to calculate a processing speed composite score.

#### 2.4.3. Executive functioning

Executive functions were measured by the NIH EXAMINER (28). NIH EXAMINER includes a composite score of five computer-based tests of working memory (dot counting, 1-back, 2-back), response inhibition (enclosed flanker), and set shifting (set shifting), and two verbally mediated tests of generativity (D-word and animal fluency). All computerized tasks included at least three practice trials.

### 2.5. Cardiometabolic outcomes

#### 2.5.1. Hemoglobin A1C

Whole blood and serum samples were collected and stored in 0.5 ml aliquots at −80 °C following baseline 12 h fasting blood draws, until used for biochemical processing. All laboratory analyses were performed by UCSF Clinical Laboratories, a CLIA-certified, CAP-accredited laboratory at UCSF Mission Bay Hospital. Hemoglobin A1C (HbA1C) levels were determined from whole blood by means of an Abbott Architect c8000 enzymatic immunoassay.

#### 2.5.2. Resting heart rate

Participant resting heart rates were measured by a clinician or study staff. A normal resting heart rate for adults ranges from 60 to 100 beats per minute (29). Elevated heart rate is a risk factor for cardiovascular morbidity and mortality (30).

#### 2.5.3. Hypertension

Personal medical history, including presence of hypertension, was collected *via* self-report during the clinical history gathering with a neurologist. If the participant did not report a history of hypertension, but indicated taking an antihypertensive drug, she/he was asked by the clinician or study staff if they were prescribed the medication for their blood pressure. If answered yes, the participant was marked as having a history of hypertension.

### 2.6. Brain MRI

#### 2.6.1. Structural neuroimaging

Magnetic Resonance Imaging (MRI) scans were performed at the UCSF Neuroscience Imaging Center using a Siemens Prisma fit 3 T scanner. Magnetization prepared rapid gradient-echo (MPRAGE) sequences were used to obtain whole brain T1-weighted images (TR/TE/TI = 2300/2.9/900 ms, *α *= 9°). The field of view was 240 mm × 256 mm, with 1 mm × 1 mm in-plane resolution and 1 mm slice thickness with a sagittal orientation.

Before processing, all T1-weighted images were visually inspected for quality control and those with excessive motion or image artifact were excluded. Magnetic field bias was corrected using the N3 algorithm (31). Tissue segmentation was performed using unified segmentation in SPM12 (32). Each participant's gray matter segmentation was warped to create a study-specific template using Diffeomorphic Anatomical Registration using Exponentiated Lie algebra (DARTEL) (33). Participants' native space gray and white matter segmentations were then normalized and modulated to study-specific template space using nonlinear and rigid-body transformations. Images were smoothed using a Gaussian kernel of 4-mm full width half maximum. Each participant's segmentation was carefully inspected to ensure the robustness of the process.

For statistical purposes, linear and nonlinear transformations between DARTEL's space and ICBM space were applied (34). Quantification of volumes in specific brain regions was accomplished by transforming a standard parcellation atlas into International Consortium for Brain Mapping (ICBM) space and summing all modulated gray matter within each parcellated region of interest (ROI) (35). Total intracranial volume was calculated for each participant as the sum of the gray matter, white matter, and cerebrospinal fluid segmentations. Medial temporal lobe volume was selected as our brain MRI outcome, as exercise engagement in older adults has previously been associated with greater volume in this particular region (36). For the purpose of this study, medial temporal lobe volumes included the following bilateral regions: hippocampus, entorhinal cortex, and the parahippocampal gyrus.

### 2.7. Statistical analyses

First, we examined associations among the four PA measures (Fitbit steps, Fitbit calories burned, PASE, CHAMPS-MET) with Spearman's rank correlations to evaluate comparability. Next, we evaluated relationships between PA measures with demographic variables of interest *via* independent samples *t*-tests, Spearman's rank correlations, and ANOVA. We tested construct validity by evaluating the relationship between each PA measure and the cardiometabolic and cognitive outcomes of interest *via* Spearman's rank correlations or independent samples *t*-tests (e.g., HTN diagnosis), as appropriate. Lastly, we examined relationships between each PA measure and medial temporal volume outcomes *via* linear regression modeling adjusting for total intracranial volume. We reported effect sizes as Spearman's correlations, Cohen's *d*, or standardized betas, as necessary.

**Figure 1:**
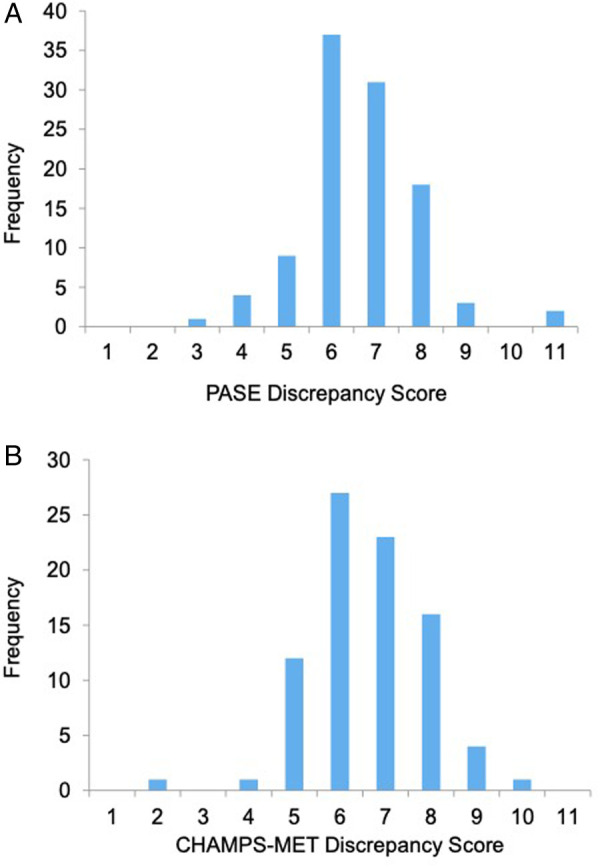
Distribution of PASE and CHAMPS discrepancy scores.

In order to identify the characteristics of older adult participants who were over or under reporting, we performed a discrepancy score analysis. Given our data indicated that Fitbit total steps demonstrated the best construct validity out of all PA measures examined, we utilized Fitbit total steps as our “gold standard” metric in these analyses. First, within participants that completed all three PA metrics (*n* = 75), we computed sample-based z-scores for each PA measure (Fitbit steps, PASE, and CHAMPS-MET). We computed individual discrepancy scores separately by subtracting Fitbit total steps z-scores from each self-report PA measure z-score (e.g., PASE or CHAMPS-MET). Distribution of discrepancy scores approximated normality (Figure 1). In this manner, higher discrepancy scores indicated greater overreporting compared to Fitbit. Next, we evaluated relationships between each discrepancy score with demographic (i.e., gender, age, education), cardiometabolic, cognitive, and brain volume outcomes. We tested relationships and group differences *via* independent samples *t*-tests, linear regression, and Spearman's rank correlations. Interpretation of effect sizes was in alignment with Cohen's *Statistical Power Analysis for the Behavioral Sciences*: coefficients of 0.10 “small,”.30 “medium,” and those of 0.50 “large” in terms of the magnitude (37).

## 3. Results

On average, participants were 74 years old, 58% female, and took 7,841 average daily steps during the monitoring period. Older age was associated with less physical activity across metrics, but only reached significance for Fitbit outcomes (Table 2). Females had lower levels of quantified physical activity for Fitbit daily steps [*t*(177) = 2.43, *p* = 0.02], Fitbit daily calories [*t*(177) = 15.77, *p* < 0.001], and lower reported physical activity for CHAMPS-MET [*t*(83) = 2.98, *p* < 0.001], but not PASE [*t*(103) = 0.72, *p* = 0.47]. Education did not meaningfully associate with the physical activity metrics (Table 2).

**Table 2:** Correlations between physical activity measures, age, and education.

	1. Age	2. Education	3. Fitbit steps	4. Fitbit calories	5. PASE
1. Age					
2. Education					
3. Fitbit steps	−0.36*	0.02			
4. Fitbit calories	−0.38*	0.13	0.50*		
5. PASE	−0.13	−0.11	0.35*	0.20*	
6. CHAMPS-MET	−0.03	0.04	0.20	0.31*	0.44*

Note. *Statistically significant at *p* < 0.05.

### 3.1. Associations among physical activity measures

All of the PA metrics were positively correlated, though demonstrated only small-to-medium effect sizes (Table 2). Greater Fitbit step count was more strongly associated with PASE compared to CHAMPS-MET, whereas Fitbit calories burned was more strongly associated with CHAMPS-MET compared to PASE scores. Self-reported CHAMPS-MET and PASE scores demonstrated a medium effect size correlation with one another.

### 3.2. Cognitive outcomes

Higher Fitbit step count [*ρ*(132) = 0.28, *p *< 0.001] and calories burned [*ρ*(132) = 0.23, *p* = 0.01], but not PASE [*ρ*(78) = −0.10, *p* = 0.39] were associated with better performances on measures of executive functioning. Unexpectedly, CHAMPS-MET demonstrated an inverse relationship with executive functioning [*ρ*(63) = −0.41, *p *< 0.01]. Only Fitbit measures demonstrated expected positive associations with processing speed, though effect sizes were small (*ρ* range = 0.05–0.07, *p*s < 0.43). Fitbit step count [*ρ*(124) = 0.20, *p* = 0.03], but not Fitbit calories burned [*ρ*(124) = −0.08, *p* = 0.38], PASE [*ρ*(76) = −0.26, *p* = 0.02], or CHAMPS-MET [*ρ*(62) = −0.31, *p* = 0.02] was associated with better scores on tests assessing memory.

### 3.3. Cardiometabolic outcomes

**Figure 2:**
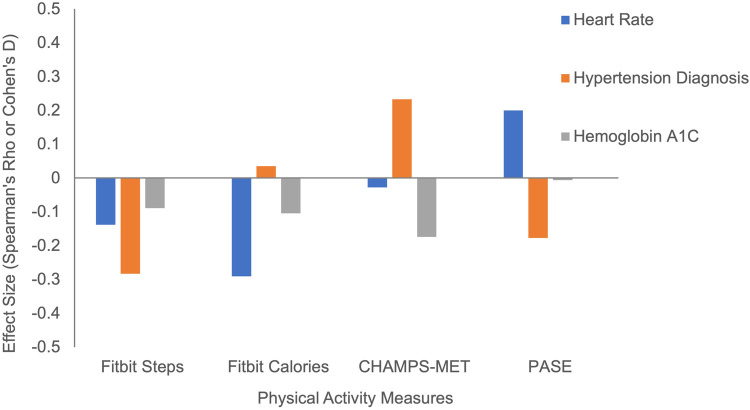
Physical activity measures with cardiometabolic outcomes. Note. *Statistically significant at *p* < 0.05.

Lower resting heart rate was significantly associated with greater daily calories burned (*ρ* = −0.29, *p *< 0.001), but less strongly with daily steps (*ρ* = −0.14, *p* = 0.08) and showed minimal associations with reported PA as measured by the PASE (*ρ* = 0.02, *p* = 0.85) or CHAMPS-MET (*ρ* = −0.03, *p* = 0.80) (Figure 2). Greater Fitbit step count (*t* = 3.058, *p* < 0.001), but not Fitbit calories burned (*t* = −0.37, *p* = 0.71), PASE (*t *= 1.43, *p* = 0.16) or CHAMPS-MET (*t* = −1.75, *p* = 0.09) was associated with a lower likelihood of hypertension. Each PA measure demonstrated expected negative associations with hemoglobin A1C that did not reach statistical significance (*ρ* range = −0.01 to −0.17, *p*s > 0.05).

### 3.4. Brain MRI outcomes

**Figure 3:**
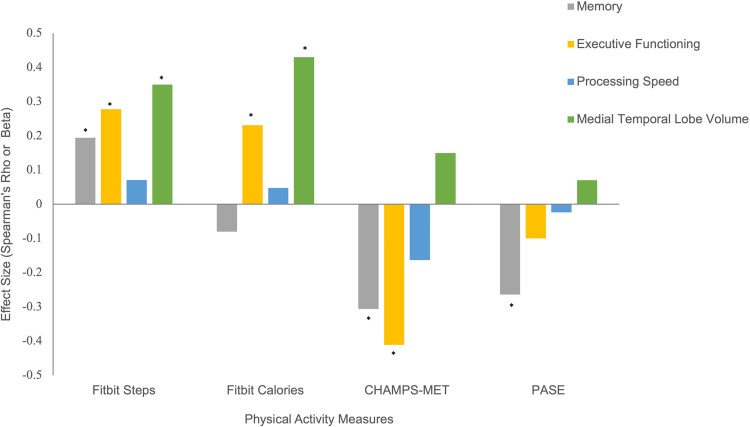
Physical activity measures with cognitive and brain MRI outcomes. Note. *Statistically significant at *p* < 0.05.

Lastly, greater Fitbit step count (*β* = 0.35, *p *< 0.001) and calories burned (*β* = 0.43, *p *< 0.001), but not PASE (*β* = 0.15, *p* = 0.17) or CHAMPS-MET (*β* = 0.07, *p* = 0.56), were associated with larger medial temporal lobe volumes (see Figure 3).

Given there were sex and age-related differences, we ran models adjusting for these factors and the patterns remained the same, with the exception of the relationship between Fitbit calories burned and memory, which showed a positive association (*β* = 0.31, *p* = 0.07)

### 3.5. Discrepancy scores

**Figure 4:**
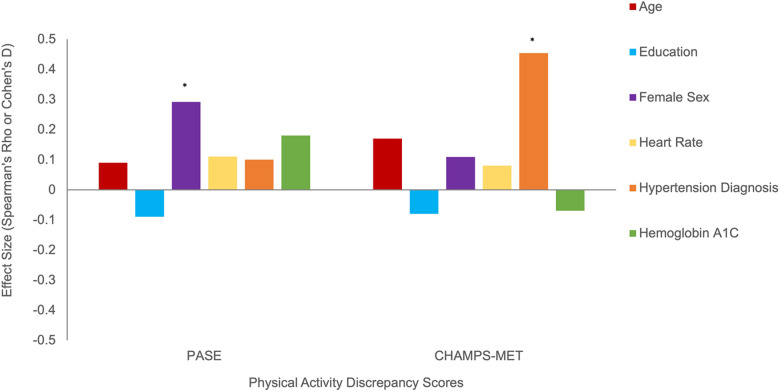
Demographic and cardiometabolic correlates of physical activity overreporting positive discrepancy score indicates greater overreporting). Note. *Statistically significant at *p* < 0.05.

**Figure 5:**
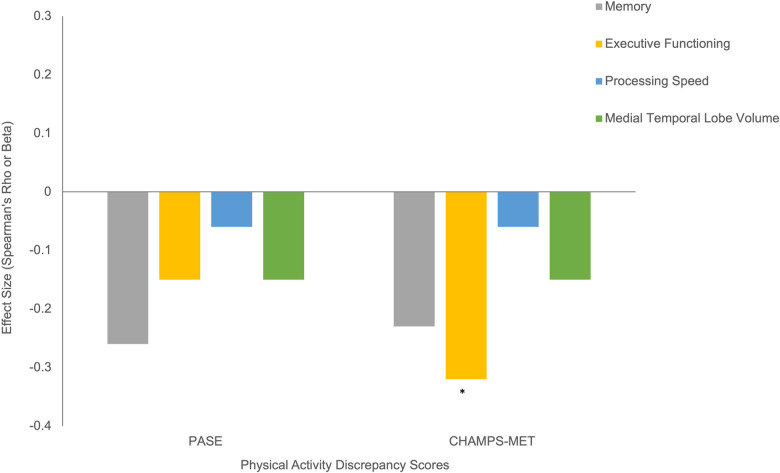
Cognitive correlates of physical activity overreporting (positive discrepancy score indicates greater overreporting). Note. *Statistically significant at *p* < 0.05.

Using Fitbit step count as the standard, discrepancy score analysis showed that females tended to overreport to a greater degree on both self-report PA scales, though this effect reached significance only for PASE (PASE Cohen's *d* = 0.61, *p* = 0.02; CHAMPS Cohen's *d* = 0.22, *p* = 0.45), see Figure 4. Older age and overreporting showed small associations that did not reach significance (*ρ* range = 0.09–0.17, *p*s > 0.14). Additionally, those with a diagnosis of hypertension overreported on CHAMPS-MET (PASE Cohen's *d* = 0.20, *p* = 0.45; CHAMPS Cohen's *d* = 1.02, *p* < 0.01), see Figure 4. Generally, worse cognition was associated with greater degree of overreporting particularly for measures of memory (*ρ* range = −0.23 to −0.26, *p*s = 0.05–0.09) and executive functioning (PASE *ρ* = −0.15, *p* = 0.28; CHAMPS *ρ* = −0.32, *p* < 0.01), see Figure 5. Decreased medial temporal lobe volume showed small associations with increased overreporting, though they did not reach statistical significance (*β* range = −0.14 to −0.15 *p*s > 0.19), see Figure 5.

## 4. Discussion

We build on previous findings that wearable, objective indicators of PA demonstrate greater overall construct validity compared to self-report measures now extending to older adults, and further detail the characteristics that may impact reporting styles on PA measures. We also highlight several novel findings on the limited interchangeability of PA metrics. Both Fitbit steps and calories burned demonstrated more consistent convergence with relevant outcomes of interest when compared to subjective measures. These results are consistent with prior studies in younger adults showing closer approximation of measuring physical activity with objective monitors in comparison to self-report (38, 39). Within Fitbit metrics, step count particularly demonstrated stronger and more consistent expected associations with each cardiometabolic, cognitive, and brain volume outcome of interest compared to calories burned, consistent with previous studies (11). These findings converge with previous literature that has demonstrated distinct associations between physical activity and executive functioning, memory, and MTL volume (36, 40, 41). While we again identify that objective digital health measures show preferable construct validity over subjective measures of physical activity, objective measures still demonstrated substantial variability with expected outcomes of interest (e.g., hypertension diagnosis, memory) and more work is warranted to elucidate factors that contribute to variability in physical activity metrics for older adults.

Notably, the associations found among the four physical activity metrics used in this study suggest a lack of cohesion within the field's current standard measurement approaches. For instance, our results demonstrated only modest relationships across the four physical activity measures (i.e., *r* = 0.2–0.30) indicating small variance explained between each measure (i.e., *R*^2^ = 4%–9% variance explained). These data suggest only minimal overlap among metrics that were created to quantify the same construct. There was also variation between Fitbit metrics and their associations with the self-report measures. Fitbit step count demonstrated a greater association with subjective reports of physical activity measured by the PASE, whereas Fitbit calories was more strongly associated with physical activity measured by CHAMPS-MET. These findings illustrate an important implication. While these subjective and objective measures were intended to capture the same construct, there is clearly variability in how each measure assesses physical activity. However, it is worth noting that CHAMPS-MET appears to be more closely related to the construct of calories burned, as expected. It is important to continue to clarify what each measure is assessing (with regard to physical activity) in order to pinpoint what factors of physical activity are beneficial in the context of brain and age-related health.

In addition to variation across the physical activity measures, our results also indicated considerable heterogeneity with associations between each activity measure and outcomes of interest. For example, Fitbit step count showed a stronger relationship to decreased likelihood of hypertension, while Fitbit calories demonstrated a stronger association with lower resting heart rate. Medial temporal lobe volume, and hemoglobin A1C demonstrated consistent, expected associations with each measure of PA; however, effects were small and often did not reach statistical significance (with the exception of Fitbit metrics and brain volume). Overall, there was notable variability in the strength and direction of examined relationships, particularly between self-reported physical activity measures and each outcome. This again suggests that physical activity may be comprised of several constructs that are differentially tapped into by self-report questionnaires and wearable devices, and/or there is imprecision when capturing physical activity across measurement tools. These findings are particularly relevant in scientific research when comparing across studies, as these objective and subjective measures do not appear to be equivalent to one another. Our results revealed that metrics capturing physical activity intensity *via* calories burned (e.g., Fitbit calories, CHAMPS-MET) may not be interchangeable with metrics of overall movement (i.e., Fitbit steps, PASE). Our data highlight how challenging measurement of physical activity can be, and that there is still room for improvement, even within “gold-standard” objective measures.

Fitbit steps demonstrated the greatest construct validity. However, there are still mixed findings in current literature with regard to its validity and reliability (42). For example, one study found that Fitbit total steps underestimated activity in healthy adults walking at faster treadmill speeds, but overestimated total steps at slower speeds (43). Similarly, another study found that Fitbit underestimated caloric expenditure in comparison to CHAMPS (39). The results of the current study, in conjunction with the variability in results from previous studies examining physical activity measures, highlight the importance of improving the current standard measurements of physical activity. In addition, these inconsistencies point to a need for refinement of the operational definitions associated with each of these physical activity measures to best understand what they are evaluating.

Notably, more precise measurement and specification of the broad range of physical activity constructs currently being utilized would allow for greater understanding of the specific movement patterns that are most critical for brain health. To date, there is not strong evidence for a particular movement (e.g., walking) or intensity (calories burned, heart rate during exercise) that is most impactful for brain health trajectories. For example, in a meta-analysis of exercise randomized control trials (RCTs) in older adults, type of exercise was not a significant predictor of cognitive benefit (44). Indeed, activities ranging from tai chi to jogging demonstrated comparable benefit. Some of the earliest evidence linking physical activity with cognitive aging demonstrated beneficial effects even with low impact activities, such as walking (45). However, several epidemiologic and RCTs indicate that cardiorespiratory activities aimed at increasing VO_2_ max may be particularly beneficial for cognitive outcomes and future dementia risk (2, 46, 47). Nonetheless, more work needs to be done in this area to understand what particular aspects of physical activity are most important for brain health.

Because of the degree of variability found within associations between measures of physical activity and important outcomes, we elected to more closely examine which factors could be driving these relationships. More specifically, we leveraged a discrepancy score analysis to identify factors that characterize participants who may overreport on physical activity questionnaires. The results demonstrated a greater degree of overreporting in females, particularly for the PASE. This finding may impact the ability of studies to determine sex-related differences in physical activity for brain and age-related health. In addition, participants who were most discrepant generally had higher levels of physical activity across all measures, which may suggest that self-report measures are less accurate in detecting physical activity levels for those who are very active.

We also found that individuals who engaged in the greatest degree of overreporting were older adults who performed worse on cognitive assessments, suggesting subjective measures may be systematically confounded by cognitive ability when assessing activity level in the aging population. This finding converges with prior studies demonstrating that utilization of self-report measures in older adults for measuring physical activity is less accurate (48). In addition, cognitive functioning declines with age as a group, which in turn may increase risk of inaccurate responses on self-report measures (49). In addition to decline in cognition, a greater degree of overreporting was also associated with smaller medial temporal lobe volume. Generally, our findings demonstrated that individuals with poorer vascular and cognitive health tended to overreport to a greater degree. These novel findings increase our understanding of possible factors that could be contributing to reporting bias in older adults, namely their vascular and cognitive health. Future studies should examine whether these discrepancy scores could be used to predict individuals at risk for adverse brain aging.

Our study is not without limitations. Simply wearing a Fitbit may have increased participants' motivation to move, which could lead to possible a possible confound in our data. However, we noted that all feedback from the Fitbit was removed from the device so participants were otherwise blinded to real time activity levels. Our sample was not representative of the general population, with 85 percent of participants identifying as White, limiting generalizability. Furthermore, our sample was relatively small and had some variability across measures, which may have biased the outcomes. There are also inherent biases that can occur when utilizing digital technology with older adults; notably, data collection is contingent on successful device use, which may be impacted by technology familiarity. Therefore, study findings have limited generalizability to older adults with low levels of technology literacy. In addition, the high educational attainment of this cohort also impacts our generalizability and is particularly important considering higher education has been associated with lower risk of developing neurodegenerative disease. It is possible that our results are limited by a slight gap in time between obtaining self-report data and gathering metrics from Fitbit monitors. At the visit, participants reported physical activity from the last seven days *via* PASE, and from the prior 30 days *via* CHAMPS. Fitbit monitoring took place in the following 30 days post visit. However, physical activity levels are a generally stable trait (50), so it is unlikely this time discrepancy significantly affected our findings.

Our study is also limited by the lack of other “gold-standard” objective measures of physical activity and fitness, such as the 6-min walk or a research-grade accelerometer (e.g., Actigraph GT3X+). With these other objective measures, we could have more comprehensively examined Fitbit as an actigraphy metric. In addition, a longitudinal study design would have provided the opportunity to understand the reliability of subjective and objective PA measures over time and meaningfully track changes in cognitive and vascular health related to physical activity.

Without precision and specificity, it is difficult to pinpoint which aspects of physical activity contribute to brain health. Our results suggest that objective quantification of physical activity demonstrates the best validity and high clinical relevance. Moreover, issues regarding reporting bias may be especially important in older adults with lower vascular, cognitive and brain structural statuses. These findings also begin to broaden our understanding of what physical activity metrics represent to facilitate better-informed recommendations for healthy aging.

## Data Availability

The raw data supporting the conclusions of this article will be made available by the authors, without undue reservation.
